# Effects of the Topical Application of Hydroalcoholic Leaf Extract of *Oncidium flexuosum* Sims. (Orchidaceae) and Microcurrent on the Healing of Wounds Surgically Induced in *Wistar* Rats

**DOI:** 10.1155/2011/950347

**Published:** 2011-06-09

**Authors:** Fernanda Oliveira de G. de Gaspi, Mary Ann Foglio, João Ernesto de Carvalho, Gláucia Maria T. Santos, Milene Testa, José Roberto Passarini, Cristiano Pedroso de Moraes, Marcelo A. Marreto Esquisatto, Josué S. Mendonça, Fernanda A. Sampaio Mendonça

**Affiliations:** ^1^Núcleo de Ciências da Saúde do Centro Universitário Hermínio Ometto (UNIARARAS), Av. Dr. Maximiliano Baruto, 500, Araras, CEP: 13607-339, SP, Brazil; ^2^Centro Pluridisciplinar de Pesquisas Químicas Biológicas e Agrícolas (CPQBA), UNICAMP, Campinas, SP, Brazil; ^3^Departamento de Clínica Médica, Faculdade de Ciências Médicas da Universidade Estadual de Campinas (UNICAMP), Campinas, SP, Brazil; ^4^Programa de Pós Graduação em Ciências Biomédicas, Centro Universitário Hermínio Ometto (UNIARARAS), Araras, SP, Brazil; ^5^Hospital São Lucas, Diadema, São Paulo, SP, Brazil

## Abstract

This study evaluated the wound healing activity of hydroalcoholic leaf extract of *Oncidium flexuosum* Sims. (Orchidaceae), an important native plant of Brazil, combined or not with microcurrent stimulation. *Wistar* rats were randomly divided into four groups of nine animals: control (C), topical application of the extract (OF), treated with a microcurrent (10 *μ*A/2 min) (MC), and topical application of the extract plus microcurrent (OF + MC). Tissue samples were obtained 2, 6, and 10 days after injury and submitted to structural and morphometric analysis. The simultaneous application of OF + MC was found to be highly effective in terms of the parameters analyzed (*P* < .05), with positive effects on the area of newly formed tissue, number of fibroblasts, number of newly formed blood vessels, and epithelial thickness. Morphometric data confirmed the structural findings. The *O. flexuosum* leaf extract contains active compounds that speed the healing process, especially when applied simultaneously with microcurrent stimulation.

## 1. Introduction

Medicinal plants have been used since ancient times for the treatment of various skin and dermatological disorders, especially cuts, wounds, and burns [1]. Current phytotherapics agents have also been shown to be highly effective in the treatment of these diseases [2–4]. *Oncidium flexuosum *Sims. (family Orchidaceae), popularly known as the “dancing doll orchid,” is one of the most important species of the *Oncidium* genus in Brazil [5, 6]. This plant is an epiphyte that can reach a height of 1 m and possesses a large number of flowers that form branched inflorescences of attractive medium-sized flowers. The sepals and petals are yellow, with brown-reddish transverse stripes, and the lip is highlighted with small red dots [7, 8]. The Orchidaceae family is one of the largest angiosperm families, and more than 25,000 species have been described so far [9, 10], including important species with medicinal potential [11]. In traditional medicine, the species of this family have been used for the treatment of abscesses, inflammation, wounds, external bleedin, and chapped skin [12, 13]. 

Wound healing constitutes a complex, dynamic, and well-orchestrated process that is activated whenever disruption of skin tissue occurs. The wound healing process is characterized by a broad spectrum of events, including platelet aggregation and activation of the coagulation cascade, inflammatory infiltration, cell differentiation, and tissue remodeling. Although the cascade of these events seems to be well discriminated and can be divided into three phases, namely, inflammation, proliferation, and wound contraction and remodeling, these phases actually overlap and continued or renewed tissue damage may reinitiate the sequence at affected sites within an existing wound. The application of low-amperage electrical stimuli modifies the healing process in living organisms, especially factors that delay or impair this process [14–16]. Electrical stimulation of different amplitudes and frequencies has been shown to promote modifications in cell and tissue responses in experimentally induced injuries [17–19] and can be used to promote tissue repair. Stimulation of live cells with low-intensity electrical currents directly affects the membrane potential and is associated with changes in ion gradients across the cell membrane, causing an increase in the synthesis of ATP followed by increased protein synthesis [14, 19, 20]. Numerous taxonomic reviews of the *Oncidium* genus are available in the literature [7–9], but there are no scientific data regarding the pharmacological activities of *Oncidium flexuosum* Sims. (Orchidaceae). Therefore, the scope of the present study was to evaluate the effects of leaf extract of this important Brazilian plant species combined with microcurrent stimulation or alone on wound healing.

## 2. Materials and Methods

### 2.1. Plant Material

Fresh *Oncidium flexuosum* leaves were collected in the medicinal plant garden of Uniararas (Projeto Saúde & Harmonia), Araras, São Paulo, Brazil, between December 2008 and February 2009, and identified by MSc. Cristiano Pedroso de Moraes. A voucher specimen (45341) was deposited at Herbário Rioclarense (HRCB), Instituto de Biociências, Universidade Estadual Paulista, Rio Claro/São Paulo, Brazil.

### 2.2. Preparation of the Hydroalcoholic Leaf Extract

Fresh leaves (50 g) were selected, cleaned, and macerated dynamically with 300 mL of an aqueous ethanol solution (7 : 3, v/v) for 4 h at room temperature. This procedure was repeated 3 times with the same powder and the same solvent. After filtration, the solvent was completely evaporated under vacuum at 40°C in a rotary evaporator, and the hydroalcoholic *Oncidium flexuosum* leaf extract (OF) was obtained after lyophilization [21]. The yield of the lyophilized extract was 9%.

### 2.3. Phytochemical Screening Method

The qualitative identification of the chemical constituents was carried out in the same extract as that used in the wound repair tests using chemical methods and thin-layer chromatography according to the methods proposed by Marini-Bettòlo et al. [22], Harborne [21], and Matos [23]. The dried extract (100 mg) was used for each test as described below. The presence of polyphenolic compounds was analyzed with 1% ferric chloride solution. Tannins were identified using the dried extract dissolved in water with 2 mL sodium chloride (2%), filtered, and mixed with 5 mL 1% gelatin solution. The presence of flavonoids was determined using 1% aluminum chloride solution in methanol, concentrated hydrochloric acid, magnesium turnings, and potassium hydroxide solution. Dragendorff's reagent was used to evaluate the presence of total alkaloids. Saponins were analyzed based on their capacity to produce foam. For the detection of triterpenes, the extract was mixed with 5 mL chloroform, warmed at 80°C for 30 min, and then treated with a small volume of concentrated sulfuric acid. Additionally, the extract (OF) was analyzed by thin-layer chromatography on silica gel using chloroform : methanol (98 : 2) and hexane : ethyl acetate (80 : 20) as eluent. Flavonoid components were visualized first under UV light and then by spraying the chromatographic plates separately with a solution of vanillin in sulfuric acid and ammonium vapor, followed by incubation at 100°C for 5 min.

### 2.4. Experimental Groups

Thirty-six male *Wistar* rats (*Rattus norvegicus*), each weighing 250–300 g, obtained from the Experimental Animal Center “Prof. Dr. Luiz Edmundo de Magalhães,” Uniararas, were housed individually in cages at a constant temperature (23 ± 2°C) under a 12 : 12 h light/dark cycle, with free access to food and water. No difference in the average weight or behavior of the animals was observed between the beginning and the end of the study. This study was permitted by the Ethics Committee of Uniararas (protocol number 806/2006) and was performed according to international rules considering the animal experiments and biodiversity right [24, 25].

### 2.5. Preparation of Test Samples for the Bioassay

Each animal's back was depilated 48 h before surgical intervention. After local asepsis with 0.4% chlorhexidine digluconate, the animals were anesthetized by intraperitoneal injection of xylazine hydrochloride (20 mg/kg body weight) and ketamine hydrochloride (50 mg/kg). After the position was marked with a dermographic pen and pachymeter, a 2 cm long and 0.2 cm deep surgical incision was made (with sterile surgical blade marked previously) through the full thickness of the skin in the craniocaudal direction, according to methods described by Mustoe et al. [26] and Mendonça et al. [27]. The incision was not sutured. In view of the similar genetic background of the animals [28] and following the guidelines of the Ethics Committee of Uniararas, groups of nine animals each were used: control group, C, receiving a topical application of saline solution 0.9%; OF group receiving a topical application of the extract of *Oncidium flexuosum* (60 mg/mL); MC group, treated with microcurrent (10 *μ*A/2 min), and OF + MC group, receiving a topical application of the extract of *Oncidium flexuosum* plus microcurrent (10 *μ*A/2 min), according to the protocol of Mendonça et al. [27]. A transcutaneous electrical stimulator (Physiotonus Microcurrent, Bioset, Rio Claro, São Paulo, Brazil) was used for electrical stimulation of the microgalvanic type. The applications involved the use of two metal electrodes with a spherical tip (10 mm) positioned on the wound. The treatments were started 24 h after surgical intervention and were continued daily for 10 days. From the dose-response curve using doses of 20, 40, and 60 mg/mL of the extract of *Oncidium flexuosum*, 60 mg/mL was selected from the evaluation of the cell count in the reparative tissue.

### 2.6. Collection and Preparation of Wound Samples for Structural Analysis

In 2, 6, and 10 days after the injury, three animals in each group were killed under anesthesia; the total area of the wound was removed and submitted to structural and morphometric analysis. Each sample was removed and fixed in 10% formalin in Millonig buffer, pH 7.4, for 24 h at room temperature. Next, the specimens were washed in buffer and processed for embedding in Paraplast (Merck). Longitudinal sections (7 *μ*m) were stained with hematoxylin/eosin for routine histology, with picrosirius-hematoxylin for the observation of collagen fibers. The specimens were examined and documented using a Leica DM 2000 photomicroscope at the Laboratory of Micromorphology, Centro Universitário Hermínio Ometto, Uniararas.

### 2.7. Morphometric Analysis

Cross-sections of the midregion of the experimental wound were used for the determination of the following morphometric parameters: tissue repair area (×10^3^ *μ*m^2^), total number of cells (fibroblastic and inflammatory cells) (*n*/10^3^ *μ*m^2^), number of newly formed blood vessels (*n*/10^3^ *μ*m^2^), and thickness of the regenerating epithelium (*μ*m). For this purpose, three samples were randomly selected among the sections obtained. All images were captured and digitalized using a Leica DM 2000 photomicroscope. The measurements were made on the digitalized images using the Leica Image Measure and Sigma Scan Pro 6.0 programs.

The results were compared by ANOVA and the Tukey posttest, with the level of significance set at 5%. The results were entered into spreadsheets of the Biostat for Windows XP program.

## 3. Results

### 3.1. Phytochemical Screening

Preliminary phytochemical analysis of the extract (OF) using chemical methods showed a predominance of phenolic compounds, such as flavonoids and tannins, and the presence of triterpenes. However, no total alkaloids or saponins were detected.

The best separation of the components of the hydroalcoholic extract was achieved using chloroform : methanol (98 : 2) as the mobile phase. Thin-layer chromatography also revealed the presence of polyphenols, tannins, and triterpenoids.

### 3.2. Structural and Morphometric Analysis of Wound Repair

Wound healing was studied in the different groups by comparing inflammatory processes (leukocytosis, hemorrhage and exudate), proliferative processes (fibroblastic, hyperplasia, epithelization, and angiogenesis), and tissue reorganization. This was completed in all animals within the 10-day observation period. Structural analysis was performed on days 2, 6, and 10. 

The proliferative phase was observed early on day 2 after experimental injury in animals of the groups treated with microcurrent (MC) and with the combination of microcurrent and *O. flexuosum* extract (OF + MC) when compared to the control group and group treated with extract OF alone.

**Figure 1:**
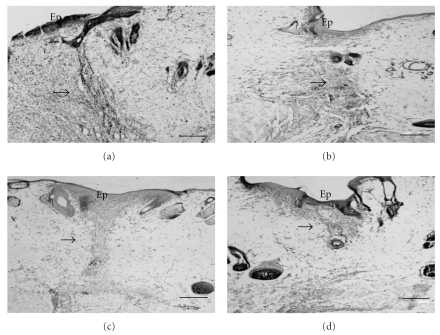
Photomicrographs of cross-sections of skin obtained from the back of *Wistar* rats on day 10 after surgically induced injury. (a) Control group; (b) group topically treated with *Oncidium flexuosum *extract; (c) group treated with microcurrent (10 *μ*A/2 min) (MC); (d) group treated with *O. flexuosum *extract plus microcurrent (10 *μ*A/2 min) (OF + MC). Ep: epidermis; (→): tissue repair area. The sections were stained with toluidine blue. Bar = 100 *μ*m.

**Figure 2:**
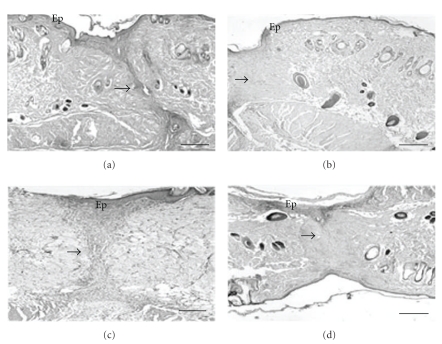
Photomicrographs of cross-sections of skin obtained from the back of *Wistar* rats on day 10 after surgically induced injury. (a) Control group; (b) group topically treated with *Oncidium flexuosum *extract; (c) group treated with microcurrent (10 *μ*A/2 min) (MC); (d) group treated with *O*. *flexuosum *extract plus microcurrent (10 *μ*A/2 min) (OF + MC). Ep: epidermis; (→): tissue repair area. The sections were stained with picrosirius-hematoxylin. Bar = 100 *μ*m.

**Figure 3:**
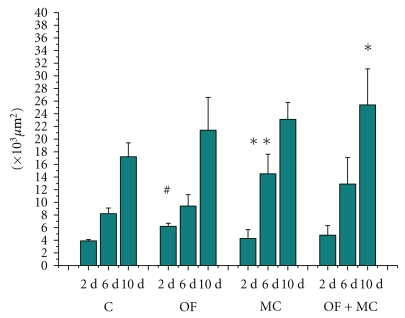
Size of the tissue repair area (×10^3^ *μ*m^2^) in the region of the experimental wound. C: control group; OF: group topically treated with *Oncidium flexuosum *extract; MC: group treated with microcurrent (10 *μ*A/2 min); OF + MC: group treated with *O*. *flexuosum *extract plus microcurrent (10 *μ*A/2 min). Samples collected on days 2 (2 d), 6 (6 d), and 10 (10 d) after injury were analyzed. The results are reported as the mean and standard deviation obtained for each group and were compared by ANOVA and the Tukey posttest (*P* < .05). Significant differences between control and treated groups in the distinct times of sampling are indicated by #, ∗∗, and ∗.

**Figure 4:**
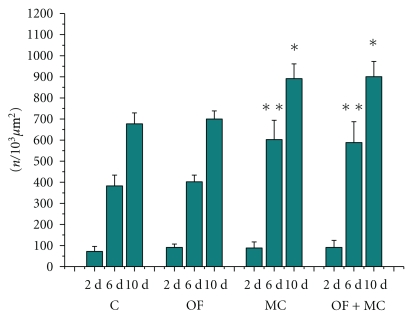
Total number of cells (*n*/10^3^ *μ*m^2^) in the region of the experimental wound. C: control group; OF: group topically treated with *Oncidium flexuosum *extract; MC: group treated with microcurrent (10 *μ*A/2 min); OF + MC: group treated with *O. flexuosum *extract plus microcurrent (10 *μ*A/2 min). Samples collected on days 2 (2 d), 6 (6 d) and 10 (10 d) after injury were analyzed. The results are reported as the mean and standard deviation obtained for each group and were compared by ANOVA and the Tukey posttest (*P* < .05). Significant differences between control and treated groups in the distinct times of sampling are indicated by ∗∗ and ∗.

**Figure 5:**
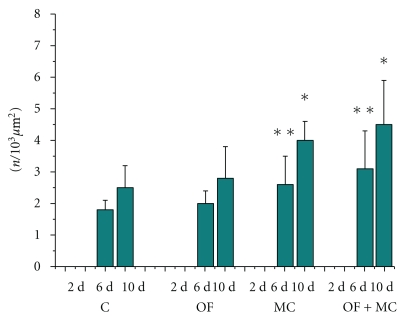
Total number of newly formed blood vessels *(n*/10^3^ *μ*m^2^) in the region of the experimental wound. C: control group; OF: group topically treated with *Oncidium flexuosum *extract; MC: group treated with microcurrent (10 *μ*A/2 min); OF + MC: group treated with *O*. *flexuosum *extract plus microcurrent (10 *μ*A/2 min). Samples collected on days 2 (2 d), 6 (6 d), and 10 (10 d) after injury were analyzed. The results are reported as the mean and standard deviation obtained for each group and were compared by ANOVA and the Tukey posttest (*P* < .05). Significant differences between control and treated groups in the distinct times of sampling are indicated by ∗∗ and ∗.

**Figure 6:**
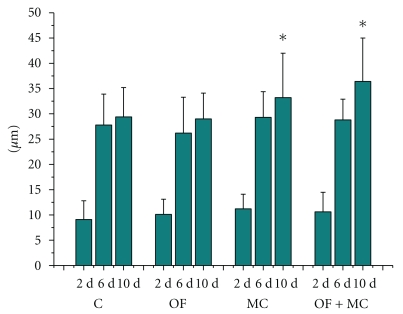
Epithelial thickness (*μ*m) in the region of the experimental wound. C: control group; OF: group topically treated with *Oncidium flexuosum *extract; MC: group treated with microcurrent (10 *μ*A/2 min); OF + MC: group treated with *O*. *flexuosum *extract plus microcurrent (10 *μ*A/2 min). Samples collected on days 2 (2 d), 6 (6 d), and 10 (10 d) after injury were analyzed. The results are reported as the mean and standard deviation obtained for each group and were compared by ANOVA and the Tukey posttest (*P* < .05). Significant differences between control and treated groups in the distinct times of sampling are indicated by ∗.

The structural characteristics of the fibrous matrix were also evaluated in the different groups. A predominance of thin and poorly compacted collagen fibers was observed in samples collected on day 6 after experimental injury in all groups. However, compact and medium-thick fibers were observed on day 10 when compared to intact tissue at the border of the wound (Figures 1 and 2). These results were supported by morphometric analysis comparing the different groups (Figures 3, 4, 5, and 6).

Two days after experimental injury, the tissue repair area was significantly greater in the OF group compared to the other experimental groups. This is the inflammatory period of repair process and probably the application of the extract containing *O. flexuosum *exerted an anti-inflammatory action favoring the repair of the lesion. A significantly greater tissue repair area was observed in the MC group as early as on day 6. On day 10 after injury, the wound area was completely reepithelized in the group treated with the combination of *O. flexuosum* extract and microcurrent, with the difference being significant when compared to the other groups (Figure 3).

With respect to the total number of cells on day 6 and 10 after injury, similar numbers were observed in the MC and OF + MC groups, which were significantly higher than those found in the control and OF groups (Figure 4).

The same pattern was observed for the total number of cells and the number of newly formed vessels. A larger number of newly formed vessels inside the wound area were observed in the OF + MC and MC groups on days 6 and 10 after experimental injury when compared to the other groups (Figure 5). 

Additionally, the thickness of newly formed epithelium was higher in the MC and OF + MC groups only on day 10 of treatment (Figure 6).

**Figure 7:**
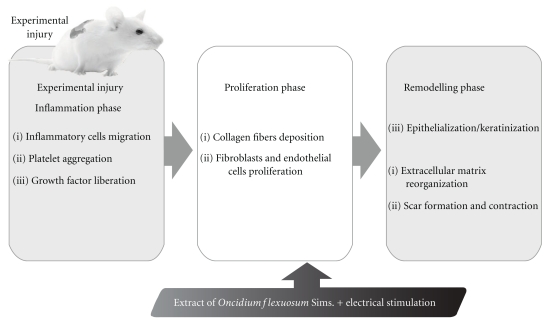
Hypothetical diagram demonstrating the possible effect of *Oncidium flexuosum *leaf extract in wound healing activity.

Structural and morphometric analysis suggested that the phytochemical content of the leaf extract of *O. flexuosum *might be responsible for collagen formation at the proliferative state, which is contributed by increased fibroblasts content (Figure 7).

## 4. Discussion

The understanding of the biological and pathological events that occur during the healing process is of the utmost importance for the treatment of wounds. Clinical evidence shows that the repair of connective, dermal and subdermal tissues can be accelerated by the external application of a low-intensity electrical current [19, 29]. Microcurrent electrical stimulation is called biostimulation since this is compatible with that of endogenous currents that act in the organism at the cellular level [14, 17, 18, 30]. This technique is not invasive, presumably has an antioxidant effect, and it showed potential to accelerate wound healing [31].

The combination of the low levels of antioxidants and raised levels of free radical played a major role in delaying wound healing in aged rats and diabetic rats [32]. Microcurrent has been used in the treatment of chronic wounds [33–35]. Lee et al. [31] used a 100 nA current 3 *μ*A in the treatment of chronic wounds and ulcers associated with chronic diseases and found that the application of such currents supposedly provides electrons to tissues saturating free radicals and facilitating tissue repair.

In the present study, it was evident from the morphometric analysis application that microcurrent alone or combined with OF was significant in promoting an increase in the total number of cells, blood vessels, and thickness of the epithelium in the damaged area in all experimental groups subjected to this treatment at 6 and 10 days after the injury. The stimulation of wound healing by low-intensity electrical currents has been reported by various investigators [15, 18, 36, 37]. Biedeback [38] proposed that transmembrane currents open voltage-controlled calcium channels in fibroblasts, causing ATP resynthesis, activation of protein kinase mechanisms to synthesize new cellular protein, and DNA replication necessary for mitotic cell division. Mendonça et al. [27] suggested that microcurrent application to tissue injuries might be used as a coadjuvant to accelerate the healing process. Variations in cell metabolism as well as fibroblast proliferation, neovascularization, and collagen deposition in the wound area have been observed after microcurrent application [39, 40].

Topical application of the *O. flexuosum* extract (OF) promoted effects similar to those observed with microcurrent therapy, but to a lesser extent. Various studies have shown the efficacy of phytotherapics agents in wound healing [41–45]. The phytochemical compounds effective in this process and which also present marked anti-inflammatory activity include flavonoids and tannins, also present in the extract OF. Additionally, these compounds exert antioxidant and antibacterial activity by interacting with a wide variety of enzymatic and biochemical systems [42, 46–48]. Moreover, phytochemical studies of species of the Orchidaceae family have shown the presence of structures which produce and store substances that are probably responsible for these pharmacological actions, such as flavonoids and terpenes [49, 50]. Fiallo et al. [50] demonstrated that flavonoids were detected in the leaves of the *Oncidium luridum* Lindl. The presence of these compounds in the Orchidaceae family is widely known [51] and was also present in the extract of *O. flexuosum*. 

Flavonoids are used for therapeutic purposes because of their anti-inflammatory, antifungal, antioxidant, and wound healing properties [52]. Moreover, flavonoids and their derivatives are known to decrease lipid peroxidation by improving vascularity and preventing or slowing down the progress of cell necrosis in *Colutea cilicica* Boiss. & Bal. [53]. Flavonoids have also been shown to enhance wound healing processes primarily owing to their antimicrobial and astringent properties, which appear to be responsible for wound contraction and an elevated epithelization rate [54]. Nayak et al. [55] observed a significant wound healing potential after topical application of *Vanda roxburghii* R.Br. extract (Orchidaceae), an epiphytic orchid, to wounds surgically induced in *Wistar* rats.

Tannins also have a role in wound healing processes, according to Bedi and Shenefelt [43] and Neto et al. [56]; these compounds precipitate proteins in damaged tissues, forming a protective lining that favors repair and reduces wound permeability and exudation.

The combination of microcurrent and the extract OF was advantageous in terms of all parameters studied when compared to the control group and to either treatment alone. Soares [57] combined the application of vitamin C and physical agents for the healing of experimental wounds and demonstrated that the combination of antioxidant agents and photodynamic or low-amperage electrical therapy accelerates wound healing. Filho et al. [58] also investigated the effects of simultaneous application of physical and phytotherapics agents to wounds using ultrasound and *Aloe vera* gel on an experimental model of induction of tendinitis in rats and demonstrated that this type of treatment is effective in terms of both skin repair and reduction of the inflammatory process. Mendonça et al. [27] showed that the simultaneous application of *Aloe vera* and microcurrent was effective in the treatment of open wounds, potentiating wound healing in *Wistar* rats. This seems to indicate a synergistic action between these two applications. Mendonça et al. [59] associated the low-power GaAlAs laser (830 nm) with aqueous solution curative of *Stryphnodendron barbatimao* in the treatment of septic wounds of sheep and found complete epithelization at 15 days by daily application of these agents combined. These reports suggest that this methodology where physical agents associated with herbal medicines is effective in the treatment of wounds.

## 5. Conclusion

The present results show that the extract of *O. flexuosum* was effective in experimental models of wound healing, accelerating this process. However, a better performance was observed when the extract was combined with microcurrent stimulation compared to the other treatments and to the control group, suggesting a synergistic action of these agents. The application of this treatment showed advantages in terms of all parameters studied.
